# Supplementary material for: Human and Mouse TRPA1 Are Heat and Cold Sensors Differentially Tuned by Voltage
Source: Cells. 2019 Dec 24;9(1):57. doi: 10.3390/cells9010057 (PMC7016720; doi:10.3390/cells9010057)
Supplement: Supplementary file 1 [file cells-09-00057-s001.pdf]

# Supplementary Figures

Sinica et al.

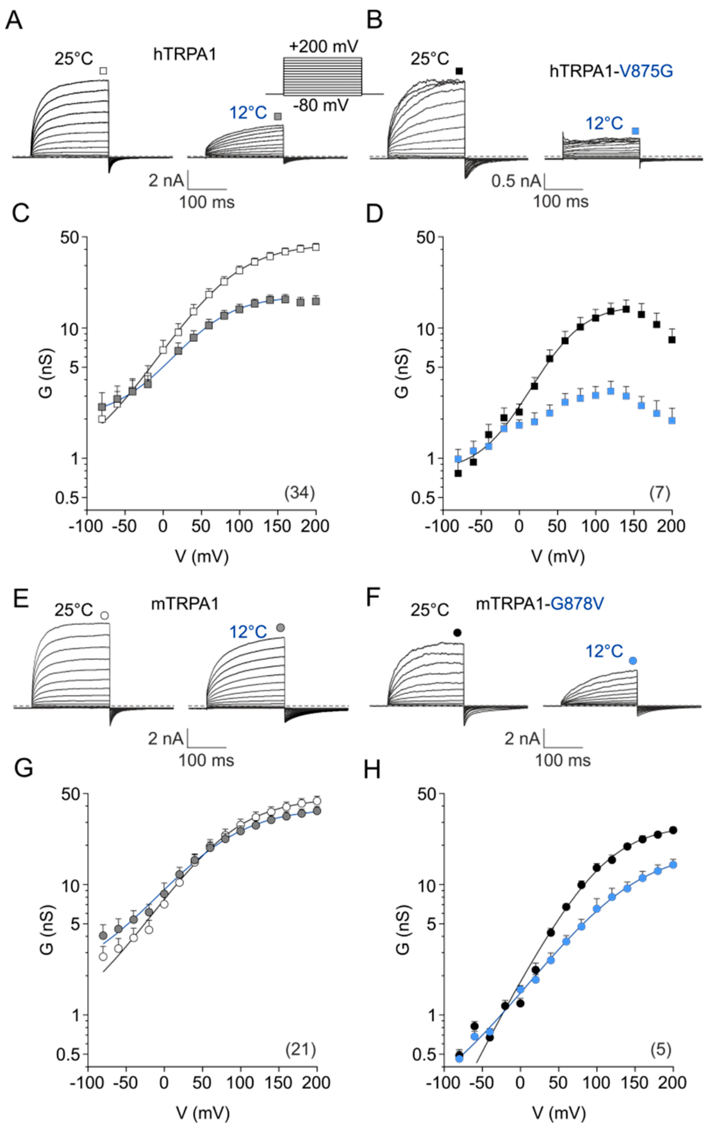


**Figure S1.** Species-specific involvement of a single residue in S5 in temperature sensitivity at close-to-saturation voltages. (**A**) Average whole-cell currents in $Ca^{2+}$-free intracellular and extracellular solutions recorded from human embryonic kidney (HEK) 293T cell line transiently expressing wild-type human TRPA1 (hTRPA1) channel, measured by indicated voltage-step protocol at 25 °C and 12 °C. Steady-state currents were measured at the end of the pulses as indicated by colored symbols atop each record. Dashed lines indicate zero current. (**B**) Average whole-cell currents of human-to-mouse mutation hTRPA1-V875G as in (**A**). (**C**) Average conductances of hTRPA1 obtained from voltage step protocols at both temperatures as in (**A**). The data represent the means + S.E.M. (*n* in brackets). The solid lines represent the best fit to a Boltzmann function as described in Materials and Methods. (**D**) Average conductances of hTRPA1-V875G obtained from voltage step protocols at both temperatures as in (**C**). The data represent the means + S.E.M. (*n* in brackets). (**E**) Average whole-cell currents through wild-type mouse TRPA1 (mTRPA1) as in A. (**F**) Average whole-cell currents of mouse-to-human mutation mTRPA1-G878V as in (**A**). (**G**) Average conductances of mouse TRPA1 (mTRPA1) obtained from voltage step protocols at both temperatures as in (**C**). The data represent the means + S.E.M. (*n* in brackets). (**H**) Average conductances of mTRPA1-G878V obtained from voltage step protocols at both temperatures as in (**C**). The data represent the means + S.E.M. (*n* in brackets).

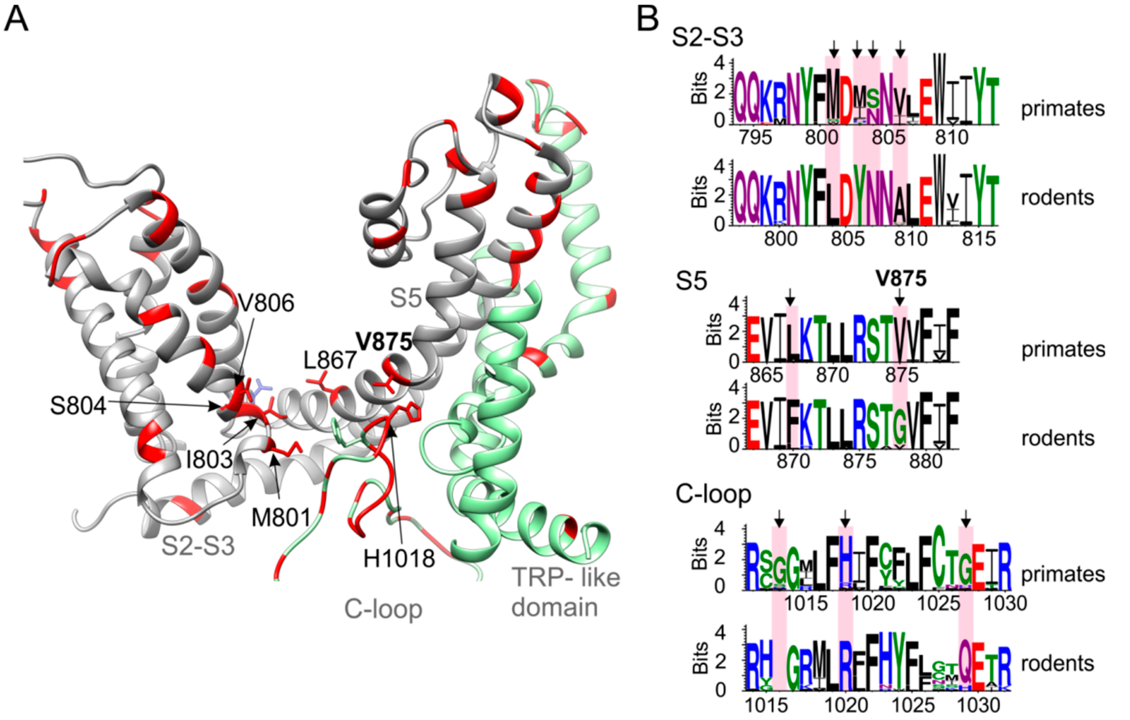


**Figure S2.** Screen for the non-homologous residues in the vicinity of V875. (**A**) Side view on the transmembrane part of the hTRPA1 model based on structure 3J9P, missing loops were modeled and fitted by molecular dynamic flexible fitting to the density map (see Materials and Methods). Two subunits (gray and green) are shown for clarity. The residues that differ from mouse orthologue are highlighted in red. The conserved residue N855, associated with familial episodic pain syndrome, is highlighted in lilac. In the C-terminal loop, side chains of H1018 and F1020 are shown. (**B**) Amino acid sequence conservation within the S2-S3, S5 and C-terminal loop regions of primate (*n* = 21) and rodent (*n* = 27) TRPA1 species represented as a sequence logo. The non-conserved residues mutated in this study are indicated by arrows.

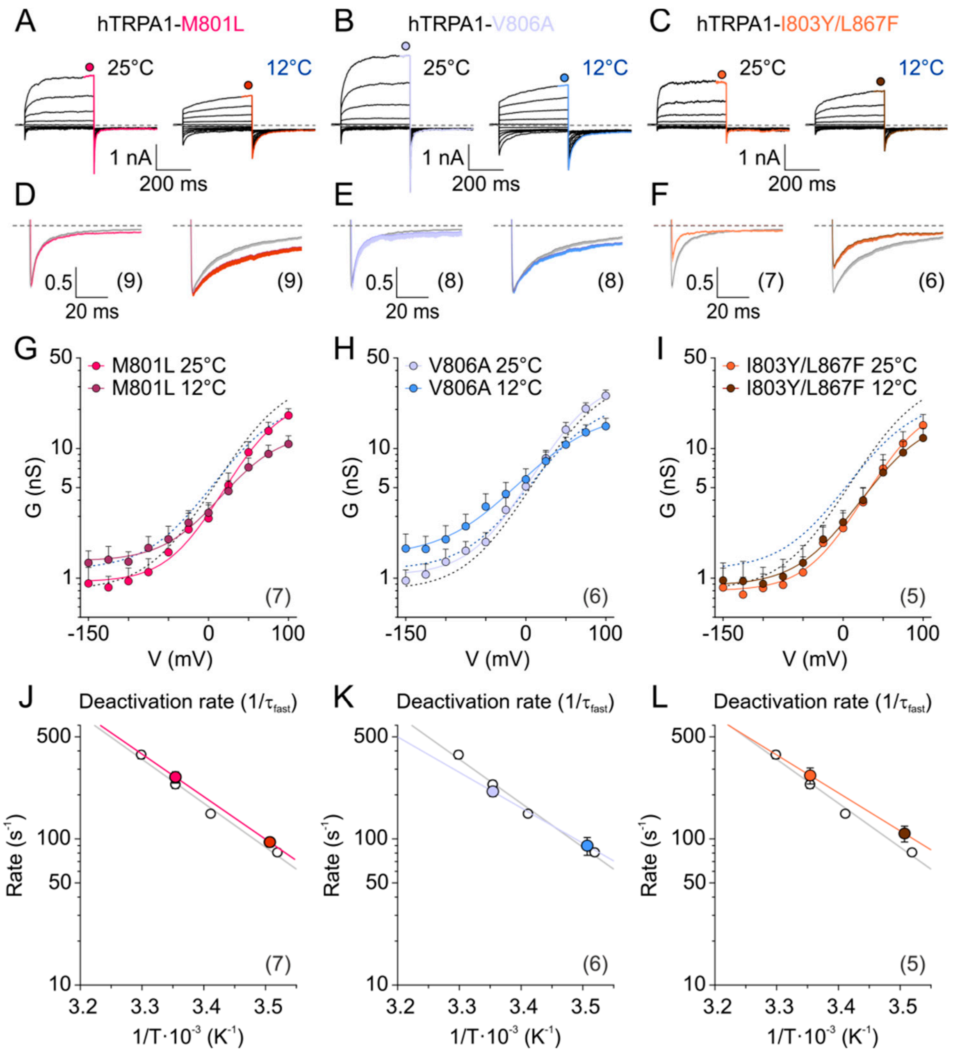


**Figure S3.** Functional screen of non-homologous residues in the vicinity of V875. (**A-C**) Average whole-cell currents in $Ca^{2+}$-free intracellular and extracellular solutions recorded from HEK293T cells expressing indicated mutants of human TRPA1 (hTRPA1), obtained by voltage step protocol as in Figure 1A at indicated temperatures**.** Steady-state currents were measured at the end of the pulses as indicated by colored symbols atop each record. Tail currents at +100 mV are colored. **(D-F)** Average tail currents following a +100mV voltage pulse from indicated mutations highlighted in (**A-C)** at 25 °C and at 12 °C. Tail currents are normalized to the maximum amplitude at +~~2~~100 mV. Colored lines and colored bars represent average normalized current –SEM (*n* indicated in brackets). Gray lines and gray bars represent wild-type hTRPA1 mean values of average normalized tail currents –SEM (*n* = 8). Dashed lines indicate zero current. **(G-I)** Average conductances of indicated hTRPA1 mutants at two different temperatures obtained from voltage step protocols as in (**A**). The solid lines represent the best fit to a Boltzmann function as described in Materials and methods. Dashed lines represent the best fit of average wild-type hTRPA1 to a Boltzmann function at 25°C (gray) and 12°C (blue). The data represent the means + S.E.M. (*n* is shown in brackets, $n \geq 6$ for wild-type hTRPA1). **(J-L)** Arrhenius plots of fast deactivation time constants of indicated mutants. White circles and gray line represent Arrhenius plot of wild-type hTRPA1 deactivation time constants. Data are shown as means ± S.E.M. (*n* of mutants indicated in brackets).

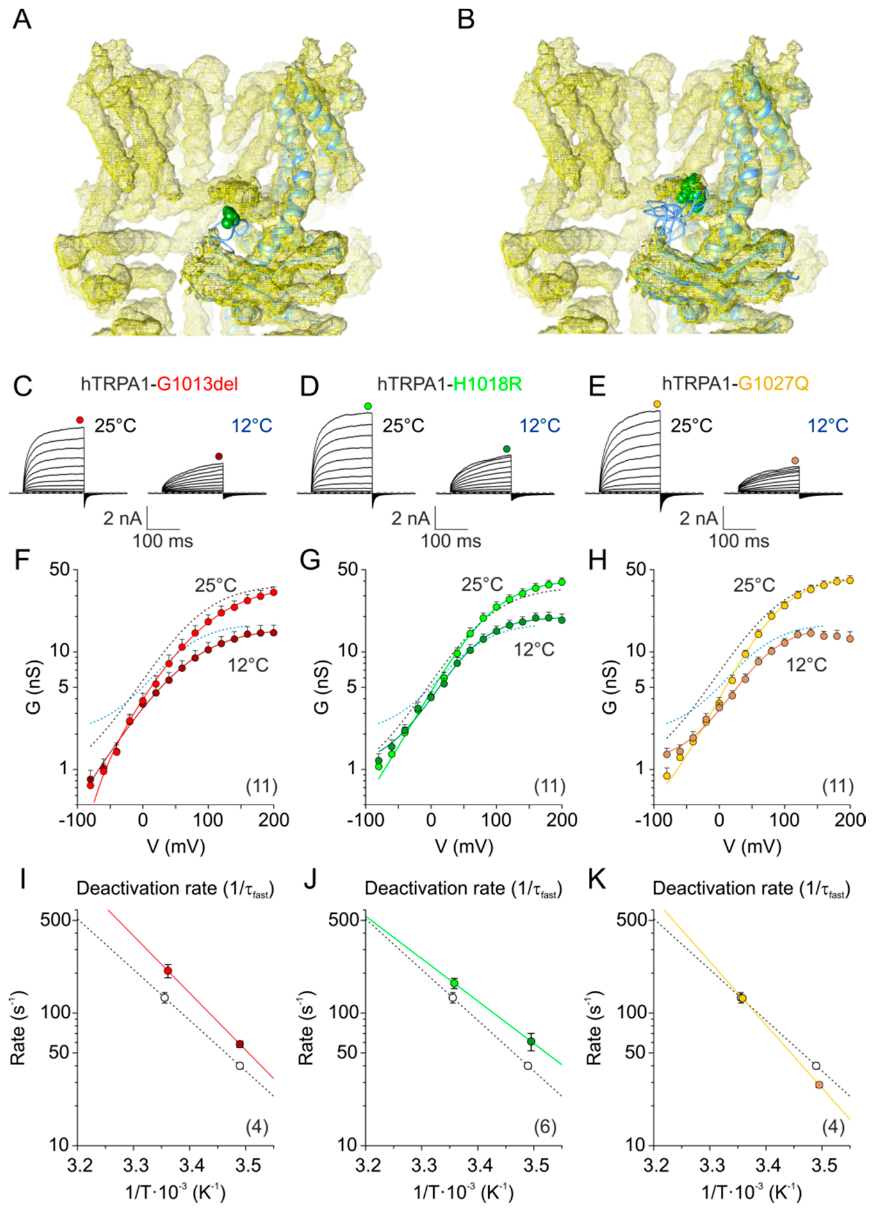


**Figure S4.** Structural-~~Functional~~ functional role of the C-terminal region. (**A**) Homology model of the C-terminal region (Y1007-R1030) with central H1018 highlighted in green. (**B**) Model of human TRPA1 with the C-terminal loop fitted to the electron density map of human TRPA1 (EMDB ID 6267). (**C-E**) Representative whole-cell currents of indicated mutants of hTRPA1, obtained by voltage step protocol as in (Figure S1A) at two different temperatures**.** Steady-state currents were measured at the end of the pulses as indicated by colored symbols atop each record. Dashed lines indicate zero current. (**F-H**) Average conductances of indicated hTRPA1 mutants at two different temperatures obtained from voltage step protocols as in (**C**). The solid lines represent the best fit to a Boltzmann function as described in Materials and Methods. Dashed lines represent the best fit of average wild-type hTRPA1 to a Boltzmann function at 25 °C (gray) and 12 °C (blue). The data represent the means + S.E.M. (*n* shown in brackets, *n* = 34 for wild-type hTRPA1). **(I-K)** Arrhenius plots of fast deactivation time constants of the mutants indicated above (in color) and the wild-type hTRPA1 (white circles and dashed gray line). Data are shown as means ± S.E.M. (*n* of mutants indicated in brackets, *n* = 20 for wild-type hTRPA1).

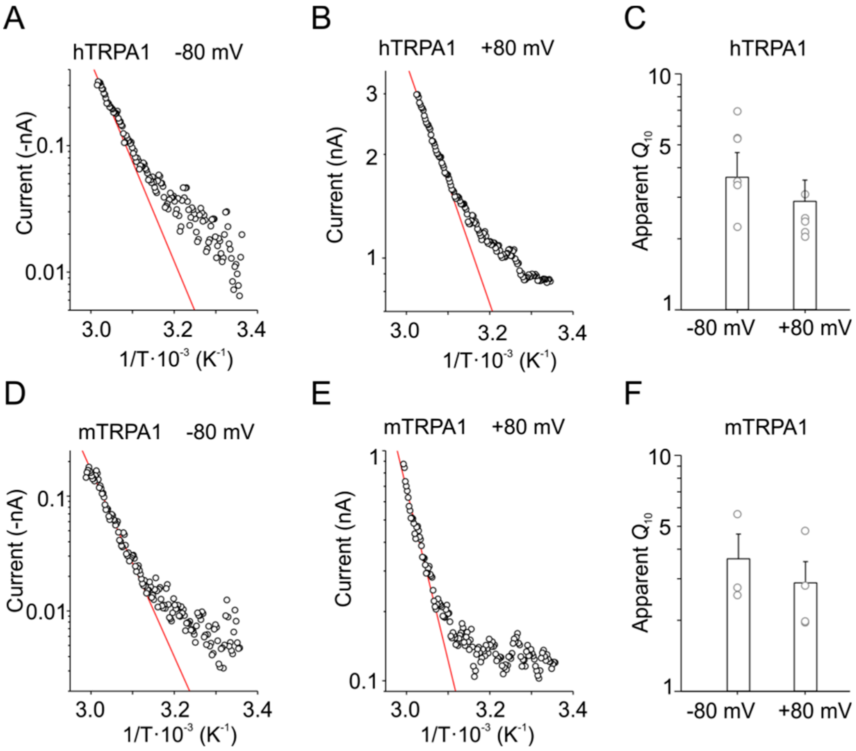


**Figure S5.** Both hTRPA1 and mTRPA1 exhibit heat-induced currents. (**A**,**B**) Arrhenius plot in which the current (*y*-axis, logarithmic scale) was plotted against the reciprocal of the absolute temperature (*x*-axis). The apparent temperature coefficients $Q_{10}$s of the hTRPA1 currents shown in Figure 5A recorded upon temperature increase, estimated by linear regression (red lines) from the slope of Arrhenius plot. The lower limit of the temperature range for estimation was defined as the temperature at which the fit declined significantly from a straight line ($r^2 < 0.98$). (**C**) Mean apparent $Q_{10}$ of hTRPA1 currents at -80 mV and +80 mV ($n = 6$ and 5). (**D**,**E**) Arrhenius plots for mTRPA1 currents at -80 mV and +80 mV, shown in (Figure 5A). (**F**) Mean apparent $Q_{10}$ of mTRPA1 currents at -80 mV and +80 mV ($n = 3$ and 4).

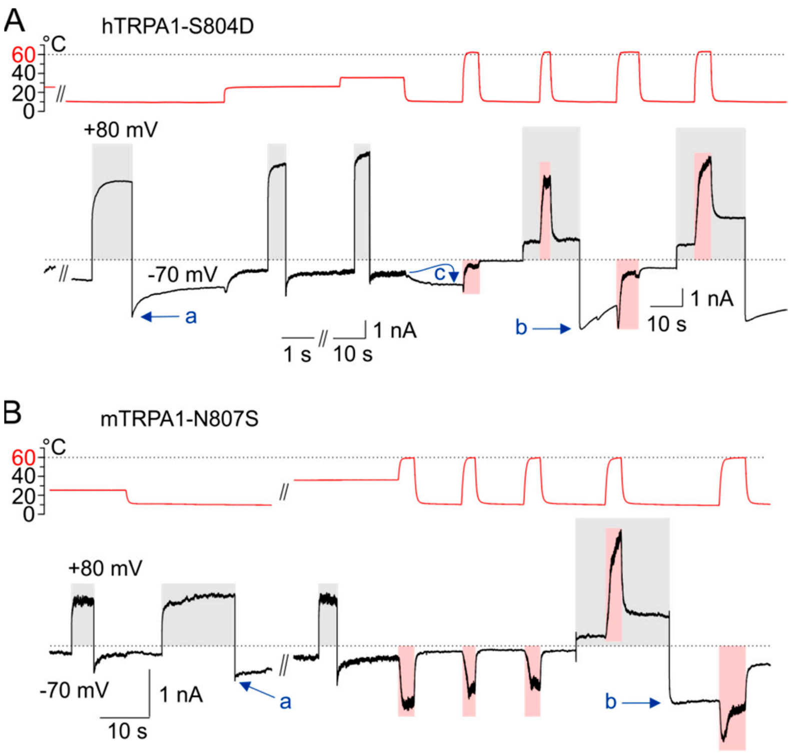


**Figure S6.** Cold-induced currents in the mutants with slowed activation/deactivation kinetics. (**A**) Representative whole-cell currents of hTRPA1-S804D in response to temperature (shaded pink area) and voltage (shaded grey area) steps. The time course of temperature changes (from 9 °C to 62 °C) is indicated above the record. Compare to wild-type hTRPA1 in (Figure 7A), where arrows a and b depict analogue situations. The temperature step from 35 °C to 9 °C elicited the 1.7-fold increase in current response (arrow c). Current to temperature relationship of the 2nd heat response is shown in (Figure ~~S7C~~S8C,D). (**B**) Representative whole-cell currents of mTRPA1-N807S as in (**A**). This mutant exhibited noxious heat evoked currents comparably large as cold evoked currents (arrow b) after concurrent voltage and temperature sensor activation at negative membrane potentials. Typical examples are shown for 3 (hTRPA1-S804D) and 3 (mTRPA1-N807S) similar recordings.

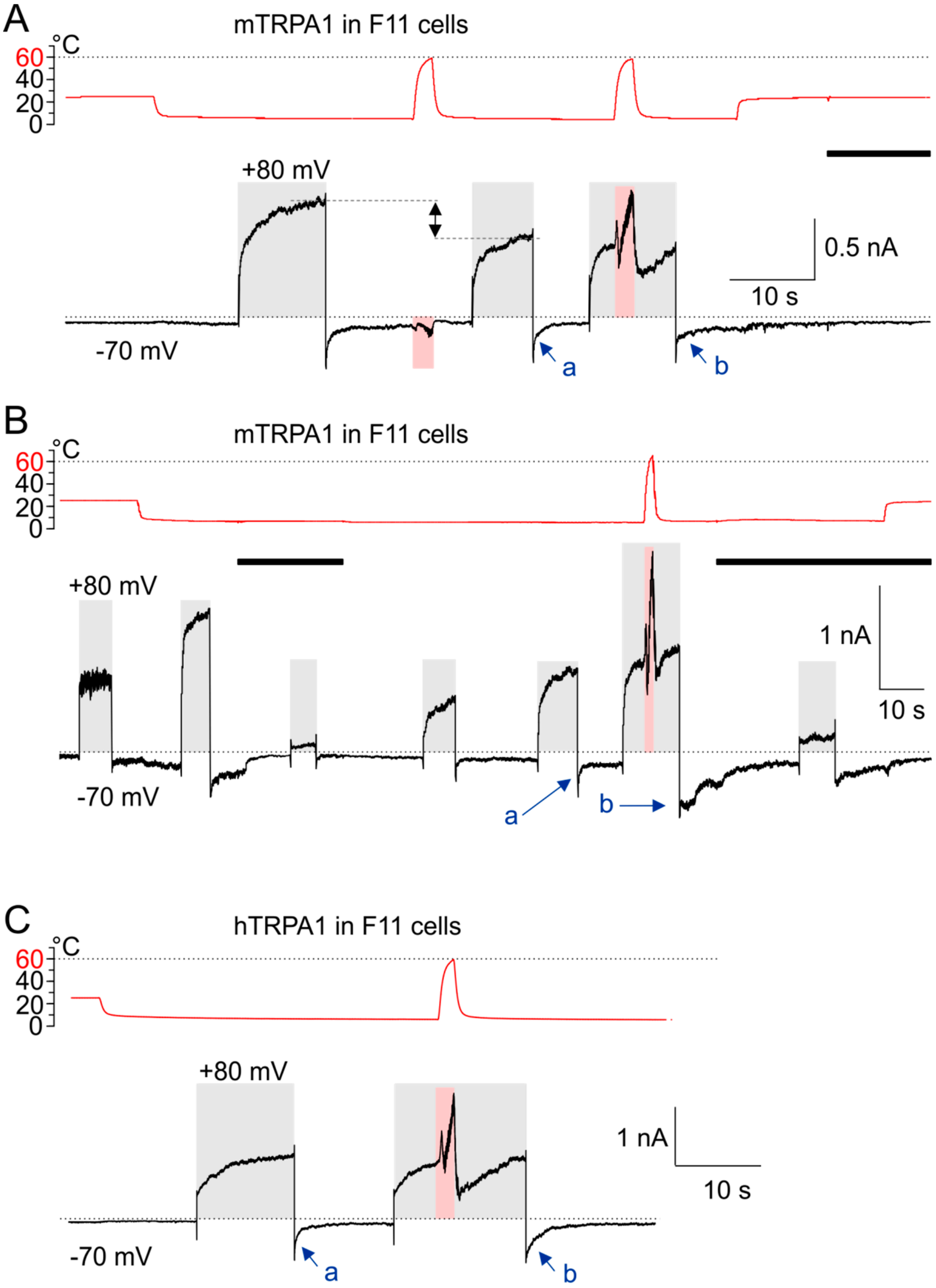


**Figure S7.** Voltage- and heat- activated currents from neuronal F11 cells expressing mouse (mTRPA1) or human (hTRPA1) TRPA1 channels. (**A**,**B**,**C**) Representative whole-cell currents recorded from neuronal F11 cells transiently expressing wild type mTRPA1 or hTRPA1 in response to temperature (shaded pink area and red traces above the measurements) and voltage (shaded grey area) steps. The exposure to 50 μM inhibitor HC-030031 is marked by black bar above traces. In (**A**), the bidirectional vertical arrow depicts analogue situation as in (Figure 6A,C). In (**A**)(**B**) and (**C**), arrows **a** and **b** depict analogue situation as in Figure 7A,C. Note a slower deactivation kinetics after concurrent activation by voltage and heat. Typical examples are shown for 8 (mTRPA1) and 2 (hTRPA1) similar recordings.

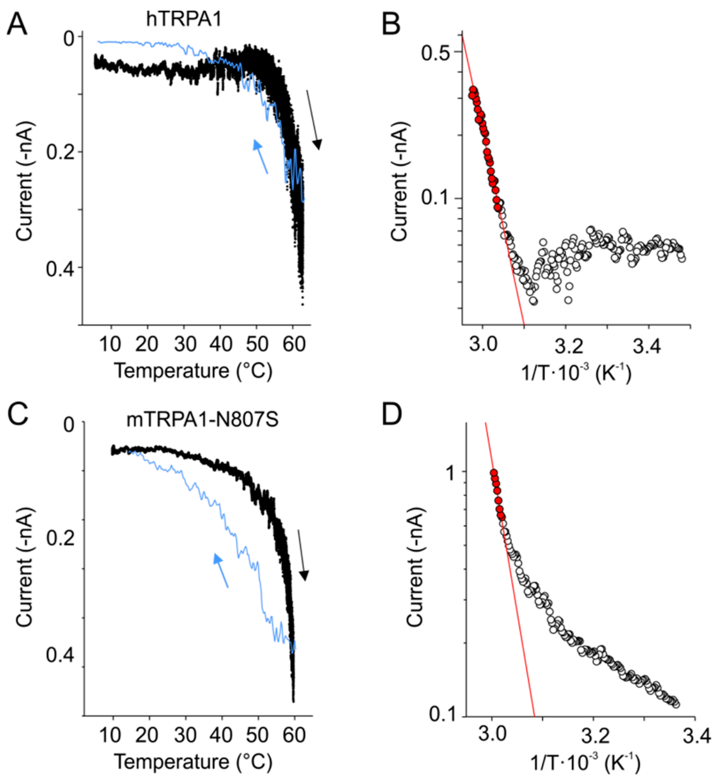


**Figure S8.** TRPA1 is directly and reversibly activated by high noxious temperatures. (**A**) Current-temperature relationship for representative whole-cell current of hTRPA1 (1st heat response shown in Figure 7A) activated by temperature step applied at a maximum speed of about 110 °C/s and at constant holding potential -70 mV. The arrows indicate the direction of temperature increase (black) or decrease (blue). The activation-deactivation is accompanied by a low degree of hysteresis. (**B**) Arrhenius plot in which the current (*y*-axis, logarithmic scale) shown in (**A**) was plotted against the reciprocal of the absolute temperature (*x*-axis). The apparent temperature coefficient $Q_{10}$ estimated by linear regression (red line) from the steepest slope of Arrhenius plot is 6.8. (**C**) Current-temperature relationship for the 2nd heat response shown in (Figure S6B). Typical examples are shown for 6 (hTRPA1) and 3 (mTRPA1-N807S) similar recordings. (**D**) Arrhenius plot of the current shown in (**C**). The apparent temperature coefficient $Q_{10}$ estimated by linear regression (red line) is 13.3.
